# The Effect of the New Lupeol Derivatives on Human Skin Cells as Potential Agents in the Treatment of Wound Healing

**DOI:** 10.3390/biom11060774

**Published:** 2021-05-21

**Authors:** Magdalena Anna Malinowska, Elżbieta Sikora, Joanna Stalińska, Jan Ogonowski, Justyna Drukała

**Affiliations:** 1 Organic Chemistry and Technology Department, Faculty of Chemical Engineering and Technology, Cracow University of Technology, 31-155 Cracow, Poland; esikora@pk.edu.pl (E.S.); jogonow@pk.edu.pl (J.O.); 2 Department of Cell Biology, Faculty of Biochemistry, Biophysics and Biotechnology, Jagiellonian University, 30-387 Cracow, Poland; joanna.stalinska@doctoral.uj.edu.pl (J.S.); justyna.drukala@uj.edu.pl (J.D.)

**Keywords:** lupeol esters, novel molecules, skin damage recovery, antioxidant mechanisms, toxicity, keratinocytes, fibroblasts, migration, proliferation, scratch assay

## Abstract

Skin barrier damage can be the result of various external factors including heat, radiation, chemicals and many others. Any interruption of the skin barrier integrity causes the exposure of the organism to harmful environmental factors. Therefore, there is an urgent need to develop novel therapeutics characterized by high bioavailability and effectiveness in skin damage recovery. Birch bark is known as a clinically proven, traditional medicinal remedy to accelerate wound healing. Lupeol, one of the main birch bark ingredients, shows a wide range of biological activity beneficial to the skin. The purpose of the research was to determine the influence of new lupeol derivatives on keratinocyte and fibroblast migration and proliferation, as well as to investigate various mechanisms of their antioxidant activity. The chemical modification of lupeol structure was intended to obtain more effective therapeutics characterized by higher bioavailability, permeability and safety of use. The novel triterpenes presented in this study were evaluated as the potential active ingredients preventing skin tissue degradation. Lupeol esters influence skin cells’ motility and proliferation. Importantly, they are able to reduce reactive oxygen species and act indirectly by protecting the skin protein structure from being oxidized by free radicals.

## 1. Introduction

The fundamental function of the skin barrier is to protect the body from harmful environmental conditions. The loss of skin integrity immediately results in various processes restoring all epidermal protective functions. The repair of wounds is an extremely complex biological process. Tissue regeneration includes migration and proliferation of keratinocytes and fibroblasts as well as angiogenesis. Old collagen is replaced, apoptosis takes place and a scar is formed. Hence, wound healing requires the integration of many complex cellular and molecular events which can be targeted at many points, leading either to accelerated or delayed healing, the later can lead to chronic wounds [1]. The increasing level of air pollution and the concentration of reactive oxygen species have a negative impact on human tissues and the process of recovery from skin damage. The theory of oxidation considers that free radicals are the major factors causing skin aging and degradation. Excessive reactive oxygen species cause collagen polymerization, which results in the impaired skin recovery process as well as the loss of skin elasticity. Plant extracts, rich sources of many active substances, such as flavonoids, terpenoids, vitamins, or enzymes are used in skin-care products as antioxidant agents. The antioxidants show various mechanisms of skin protection from oxidation processes [2]. The direct antioxidant activity ensures the chemical reduction, deactivation, of free radical forms. Indirect antioxidants, that may or may not be redox-active, are involved in a complex process as the inducers of cytoprotective proteins, so called phase 2 enzymes. As such the proteins participate in the synthesis and regeneration of direct antioxidants [2,3]. Because of high concentration of air pollutants and other destructive factors, which are nowadays a significant problem in urban areas, there is a growing tendency of rapid skin aging as well as the occurrence of skin diseases. Protein oxidation is characteristic in aging and age-related skin diseases. When the glutathione level decreases with age, the process of the skin degradation rapidly accelerates and the mechanisms for repairing the skin are slowed down significantly [4].

**Figure 1:**
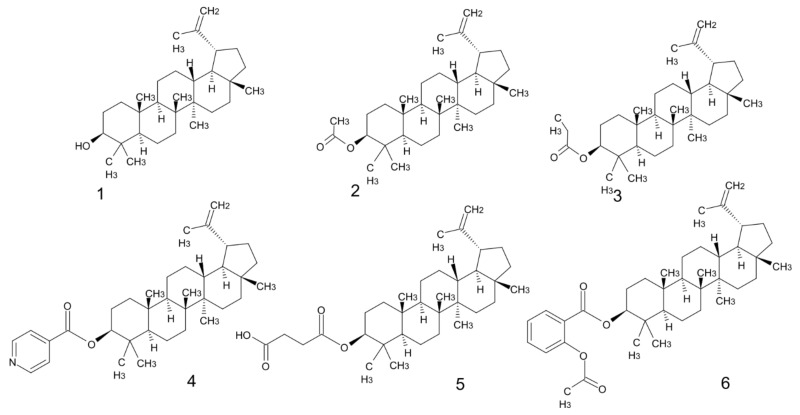
The structures of lupeol and its new derivatives (**1**: lupeol, **2**: lupeol acetate, **3**: lupeol propionate, **4**: lupeol isonicotinate, **5**: lupeol succinate, **6**: lupeol acetylsalicylate).

To meet the requirements for effective cosmetics, the active agents used should act on two levels. They should neutralize free radicals and protect skin tissues from external factors, while simultaneously inducing skin regeneration processes. Keratinocyte and fibroblast proliferation and migration are crucial in the process of wound healing, they also ensure the correct functioning of skin renewal mechanisms. Natural raw materials are a great source of effective antioxidant ingredients. The example of such promising skin-active agent is lupeol, the triterpene alcohol (Figure 1). Triterpenes not only reduce free radicals, thanks to the presence of hydroxyl group, but they act as direct antioxidants, preventing the oxidation process caused by reactive oxygen species, and what is more, they also promote local blood circulation, thus increasing oxygen supply to tissues and removing by-products of metabolism [5].

Lupeol shows a wide range of biological activities. Numerous in vitro studies confirm that lupeol can act as an antioxidant, anti-inflammatory and anti-microbial agent [6]. As a result, lupeol has been already used for many years as a remedy for skin diseases. Research concerning the application of triterpenes in cosmetics is focused in most cases on lupeol-rich plant extracts. A rich source of triterpene is white birch bark. The dry *Betula papyrifera* extract contains up to 80% of triterpenes [7] and lupeol is (after betulin) the second most available substance [8]. Another example of a lupeol-rich plant, commonly used in cosmetics formulations, is aloe vera [9]. Sticky sap from damaged leaves (called alona) is an ingredient with antioxidative, moisturizing, anti-inflammatory, anti-bacterial, anti-viral properties, it also nourishes, soothes, tightens and whitens the skin and remedies all types of irritation. Aloe stimulates the activity of fibroblasts crucial for collagen synthesis. Lupeol is also one of the triterpene compounds present in Shea butter (Butyrospermum paradoxum oil), used in cosmetic products, dedicated for skin protection against various environmental factors. This oil is applied in sun-screen, regenerating, moisturizing, skin smoothing and hair rebuilding cosmetics (shampoos, hand creams, aftershave and after-sun lotions, creams and balms) [6]. The latest studies relating to skin-care compositions, comprising a lupeol-rich extract, focus on treating and preventing connective tissue degradation [10]. They describe lupeol as an interesting active molecule for relieving skin burns and stimulating the production of high-quality type I collagen in human skin [11].

On the other hand, high lipophilicity and low water solubility of lupeol molecules influence its bioavailability and cause some limitation in its applications. Currently, various studies draw attention to the fact, that the modification of triterpenes structure, carried out with organic synthesis, can be an efficient way to enhance their activity and bioavailability [12].

The aim of our work was to determine the broad range activities of new lupeol esters (acetate, propionate, isonicotinate, succinate and acetylsalicylate). The influence of the lupeol derivatives on fibroblasts and keratinocytes proliferation and motility as well as their antioxidant activity were studied in order to determine their potential as an active ingredient of innovative skin-care products stimulating the process of wound healing and preventing the harmful effect of environmental conditions.

## 2. Materials and Methods

Five lupeol esters (acetate, propionate, succinate, isonicotinate and acetylsalicylate were obtained using an eco-friendly esterification method. In the esterification process, the commonly used hazardous reagents were replaced by less irritating conterparts [13]. This unconventional, eco-friendly, method is particularly important because the compounds obtained are potentially active substances in skin care formulations. Even trace amounts of hazardous reagents can have a toxic effect on damaged or irritated tissues. The chemical structures of the studied components: lupeol (Natchem, Cracow, Poland) and its synthesized derivatives are shown in Figure 1.

The antioxidant activity of the lupeol esters was determined using DPPH [14], ABTS [15,16] and FRAP [17] methods. The compounds capability to prevent protein damage caused by reactive oxygen species (ROS) was measured by determination of thiol and carbonyl groups concentration in albumin, used as the standard protein treated by oxidizing agent. Glutathione and gallic acid were applied as comparative antioxidative standards.

The proliferation of human keratinocytes and fibroblasts was evaluated with WST-1 assay. Migration of skin cells was determined in scratch assay test.

The chemicals used in the experiments: 1,1-diphenyl-2-picrylohydrazyl (DPPH), 2,2′-azino-bis-[3-ethylbenzotiazoline-6]-sulfonic acid (ABTS), 2,4,6-tri(2-pyridyl)-S-triazine (TPTZ), albumin, 5,5′-dithiobis-2-nitrobenzoic acid (DTNB), sodium lauryl sulfate (SLS), glutathione, gallic acid, 2,4-dinitrophenylhydrazine, Sephadex G-25 and guanidine hydrochloride were purchased from Sigma Aldrich Sp. z.o.o., Poznań, Poland. Sodium acetate, sodium phosphate, iron (III) chloride, trichloroacetic acid, hydrochloric acid and ethyl alcohol were obtained from Avantor Performance Materials Poland S.A., Gliwice, Poland. Milli-Q filtered water was obtained using water purification Millipore Simplicity system (Merck Sp. z.o.o., Warszawa, Poland).

The methods used to determination of the esters’ antioxidant capacity were based on spectrophotometric measurements. Absorbance determination were done using Nanocolor UV/VIS Spectrophotometer (Macherey Nagel GmbH & Co. KG, Dueren, Germany).

### 2.1. Antioxidant Activity

#### 2.1.1. Free Radical Scavenging Ability

Free radical scavenging activity was tested for 0.1 mol/dm^3^ ethanol solutions of the triterpenes tested. The total antioxidant activity for lupeol derivatives was determined using the modified Brand–Williams method. The ethanol solution of DPPH-1,1-diphenyl-2-picrylohydrazyl (0.00006 M) was used. The blank sample solution consisted of 3 cm^3^ of DPPH solution and 0.4 cm^3^ of ethanol. Each sample tested was prepared by mixing of 3 cm^3^ of DPPH solution and 0.4 cm^3^ of the 0.01 M triterpene ethanolic solution and then incubated for 30 min at 25 °C. The absorbances of the blank sample (A0) and the tested ones (Am) at 515 nm were measured. Each measurement was done in triplicates and the mean value of the absorbance was calculated. The total antioxidant activity was presented as an oxidation process inhibition percentage [18]. Free Radical Scavenging ability was also measured by the use of ABTS (2,2′-azino-bis-[3-ethylbenzotiazoline-6]-sulfonic acid) method [15]. The tested samples containing triterpenes were prepared as follows: to 1 cm^3^ of deionized water 0.2 cm^3^ of tested sample (0.01 M ethanolic ester solution) was added. After that 0.16 cm^3^ of ABTS 7mM aqueous solution was added to the mixture. After 7 min of incubation time, values of the samples absorbance at 734 nm were measured (Am). Simultaneously, blank sample (A0) and standard sample absorbance values were measured in the same conditions. Gallic acid was used as the standard compound. The total antioxidant activity was determined as for the DPPH method by the comparison to the blank sample.

Ferric Reducing Antioxidant Potential (FRAP) Assay was used to confirm the ability to reduce free radicals by lupeol derivatives. Gallic acid solution in ethyl alcohol (concentration 0.01 M) was used as the standard solution. FRAP solution was prepared as follows: 40 cm^3^ of acetate buffer (pH = 3.6), 4 cm^3^ of 20 mM FeCl_3_ solution and 4 cm^3^ of 40 mM TPTZ solution in 40 mM HCl were mixed and heated up to 37 °C. The test samples were prepared by adding 2.850 cm^3^ of FRAP solution to 0.15 cm^3^ of ethanolic solution of lupeol ester (concentration 0.01 M). After that, the samples were thermostated for 30 min at 37 °C. The absorbance was measured at 593 nm. The antioxidant activity was expressed in FRAP units [17]. The standard antioxidant used for all three methods was gallic acid.

#### 2.1.2. Capability to Prevent Structural Damage of Proteins

The protein-protective properties of the esters against reactive oxygen species (ROS) were also investigated. The determination of thiol and carbonyl groups in albumin structure was used to evaluate the level of protein degradation. Denaturation of protein structure is proportional to the concentration of carbonyl and thiol groups in the sample exposed to harmful reagents and conditions. In the study, protein standard (albumin) was exposed to sodium lauryl sulfate (SLS) in phosphate buffer solution. Simultaneously, samples containing lupeol esters as well as samples without these triterpenes were prepared. After that, the concentrations of thiol and carbonyl groups in samples tested were compared. Thiol groups concentration was determined using Ellmans’ reagent (5,5’-dithiobis-2-nitrobenzoic acid—DTNB). The reaction condition was alkaline (pH = 8.0) in order to ensure ionized form of thiol groups. Thionitrobenzoic anion (TNB), which is formed in the reaction between thiol group and DTNB, shows an absorbance maximum at 412 nm. Each -SH group causes the creation of one molecule of thionitrobenzoic acid so knowing the molar absorption coefficient of this compound at 412 nm (13,600 M^−1^ cm^−1^) and calculating the calibration curve using glutathione as standard, the concentration of thionyl groups in protein structure can be determined. 0.3 cm^3^ of albumin standard solution was mixed with 0.3 cm^3^ of 10% (m/m) SLS solution in 10mM sodium-phosphate buffer. Simultaneously, control sample without SLS solution was prepared. After that, 2.4 cm^3^ of buffer was added to the mixture. The samples absorbance at 412 nm was measured (A0). Next, 0.3 cm^3^ of Ellmans’ reagent solution was added to each sample except the control one (20 mg of DNTB in 50 cm^3^ of buffer). 0.3 cm^3^ of phosphate buffer was added to the control sample. Then, all samples were thermostated for 60 min at 37 °C and the absorbance was measured (A1). The absorbance difference (A1–A0) determines the concentration of thiol groups in the tested samples. The quantitative analysis of thiol groups concentration was prepared based on the molar absorption coefficient for thionitrobenzoic acid [19].

The concentration of carbonyl groups also determines the protein degradation level. In the experiment, 2,4-dinitrophenylhydrazine (DNPH) is being transformed into 2,4-dinitrophenylhydrazones of the tested compounds (DNP). DNP has an absorbance maximum of 366 nm and the value of molar absorption coefficient for this compound is 22,000 M^−1^ cm^−1^ [19]. Two samples were prepared simultaneously for each of tested triterpenes: 0.3 cm^3^ of albumin standard solution was added to 0.3 cm^3^ of 10% (m/m) trichloroacetic acid. The samples were centrifuged for 3 min (centrifuge Hettig Zentrifugen EB20, 5000 rpm). After decantation of supernatant, to one of the obtained precipitate, 1 cm^3^ of 2M HCl was added and to another of the obtained precipitate 1 cm^3^ of 0.2% 2,4-dinitrophenylhydrazine (m/m) aqueous solution was added. All samples were incubated at 37 °C, for 60 min. Next, 600 mg of guanidine hydrochloride was added to each sample and the solutions were filtered through Sephadex G-25. The absorbance values of the samples tested as well as control samples were measured at 370 nm. The concentrations of carbonyl groups in samples tested were calculated basing on hydrazone molar absorption coefficient (22,000 M^−1^ cm^−1^).

### 2.2. The Effect on the Proliferation and Migration of Human Skin Cells In Vitro

Human skin cells were isolated from skin biopsies taken from three healthy donors during plastic surgery and cultured as described previously [20]. Informed consent and Ethical Committee approval were obtained according to Polish law (No. 1072.6120.9.2017). Epidermal keratinocytes were cultured in serum-free keratinocyte growth medium KGM-Gold (Lonza Group Ltd., Basel, Switzerland). Dermal fibroblasts were cultured in Dulbecco’s modified Eagle’s medium (DMEM; Sigma Aldrich, Sp. z.o.o., Poznań, Poland) supplemented with 10% fetal bovine serum (Lonza Group, Basel., Switzerland) and 5000 U/mL penicillin and 5 µg/mL streptomycin (Sigma Aldrich, Sp. z.o.o., Poznań, Poland). The control consisted of cells grown in medium without added compounds. Vehicle control (0.1% DMSO) was also performed.

#### 2.2.1. Cell Proliferation

The proliferative activity of the cells was then analyzed using the WST-1 colorimetric assay (Roche Molecular Biochemicals, Mannheim, Germany) based on the quantification of the formazan dye produced by metabolically active cells. WST-1 tetrazolium salt is cleaved to formazan dye by cellular enzymes (mitochondrial dehydrogenases). Briefly, cells were plated at a density of 5 × 10^3^ per cm^2^. After 24 h, the medium was changed to medium containing tested compound or vehicle control and the cells were incubated for 24 and 48 h. WST-1 reagent was added (1:10 final dilution) and the plates were further incubated for 3 h (37 °C, 5% CO_2_, 95% humidity). The supernatant was then transferred to 96-well plates in duplicates and the absorbance of the samples was measured at 450 nm with the reference wavelength at 620 nm. The experiments were run in triplicates.

#### 2.2.2. Time Lapse Analysis of Movement of Individual Cells

Keratinocytes were seeded at the density of 5 × 10^4^ cells per well of a 6-well plate, 96 h before the addition of the compounds. Culture medium was replaced with fresh medium containing tested compounds or 0.1% DMSO (vehicle control). Cell traffic was recorded for 1.5 h, 90-sec step in time, starting 30 min after the addition of tested compounds, using DMI 6000B microscope (Leica). Data are presented in diagrams, in which the starting point of individual cell trajectory is in the center of the diagram. Analysis of movement parameters was performed as previously described [21].

#### 2.2.3. Scratch Assay

The scratch assay test was used to determine the effect of lupeol esters on the migration of human epidermal cells. Keratinocytes were plated at a density of 5 × 10^3^ per cm^2^ and cultured for 96 h. Then, a sterile tip for the automatic pipette was used to make a “wound” in the cell monolayer, test compounds were added and cell movement was recorded for 8 h at 37 °C and 5% CO_2_. The analysis of the wound surface change over time was made using the ImageJ (NIH) software.

#### 2.2.4. The Cell Morphology and Cytoskeleton

Cells were cultured for 24 h in the presence of tested lupeol esters or in control conditions on glass slides, then fixed with 3.7% paraformaldehyde in PBS (pH 7.2) (Sigma Aldrich Sp. z.o.o., Poznań, Poland) for 20 min at room temperature. Cells were permeabilized with 0.1% Triton X-100 (Sigma Aldrich Sp. z.o.o., Poznań, Poland). Nonspecific binding sites were blocked with 3% (*w*/*v*) bovine serum albumin in PBS (Sigma Aldrich Sp. z.o.o., Poznań, Poland) for 1 h. Next, cells were stained for 1 h with mouse monoclonal anti-Vinculin antibody (Sigma Aldrich Sp. z.o.o., Poznań, Poland) followed by incubation with Alexa Fluor 568-conjugated donkey anti-mouse IgG antibody (Invitrogen, Gdańsk, Poland), Alexa Fluor™ 488 Phalloidin (Invitrogen, Gdańsk, Poland) and Hoechst 33358 (Invitrogen, Gdańsk, Poland).

For visualization of F-actin, cells were incubated with TRITC-labeled phalloidin (in a concentration of 500 ng/cm^3^, Sigma Aldrich Sp. z.o.o., Poznań, Poland) in darkness for 45 min at 37 °C in a humidified incubator. Thereafter, the cells were observed under a Leica DM IRE 2 fluorescence microscope equipped with Nomarski interference contrast optics.

Statistical analysis was performed with GraphPad Prism 8.0 (GraphPad Software, Inc., San Diego, CA, USA) by unpaired Student’s *t*-test and multiple comparison one-way ANOVA (cell proliferation and migration) and with RStudio v. 1.2 by Fisher Least Significant Difference test (antioxidant activities). Data are presented as means with standard deviation.

## 3. Results and Discussion

### 3.1. Antioxidant Activity

#### 3.1.1. Free Radical Scavenging Ability

**Figure 2:**
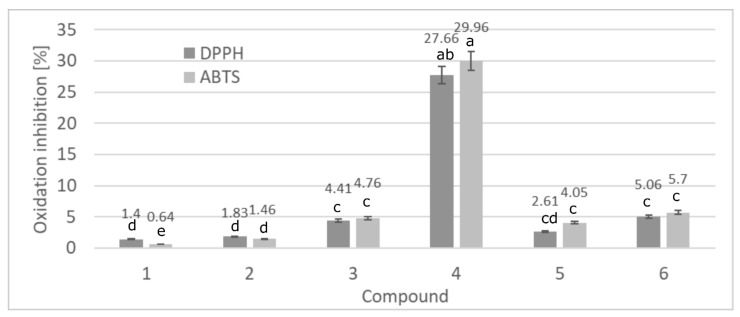
The total antioxidant activity the tested triterpenes using DPPH and ABTS methods. Numbers refer to compounds found in Figure 1 (1: lupeol, 2: lupeol acetate, 3: lupeol propionate, 4: lupeol isonicotinate, 5: lupeol succinate, 6: lupeol acetylsalicylate). Data presented as mean values ± SD (*n* = 3). Different letters (a–e) indicate significant difference between mean values at *p* < 0.001.

**Figure 3:**
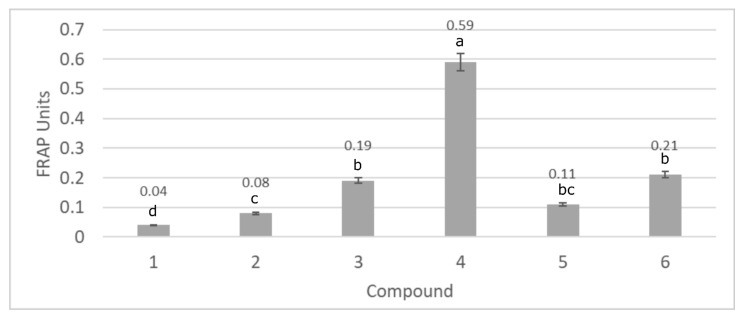
The evaluation of antioxidant activity of lupeol (1) and its new derivatives (2: lupeol acetate, 3: lupeol propionate, 4: lupeol isonicotinate, 5: lupeol succinate, 6: lupeol acetylsalicylate). Data presented as mean values ± SD (*n* = 3). Different letters (a–d) indicate significant difference between mean values at *p* < 0.001.

**Figure 4:**
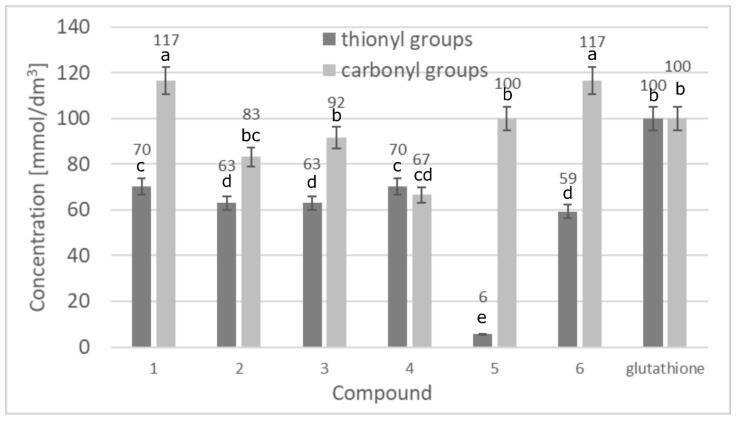
Protective activity for protein structures presented as the concentrations of thionyl and carbonyl groups, (1: lupeol, 2: lupeol acetate, 3: lupeol propionate, 4: lupeol isonicotinate, 5: lupeol succinate, 6: lupeol acetylsalicylate). Data presented as mean values ± SDstandard deviation (*n* = 3). Different letters (a–e) indicate significant difference between mean values at *p* < 0.001.

The results of antioxidant activity are presented in Figure 2, Figure 3 and Figure 4. The results are mean values from three replicates (*n* = 3) and the standard deviation was calculated (±SD). According to Fisher LSD test, the values marked by different letters are statistically different at *p* < 0.001. The ability of free radicals scavenging is crucial in the direct protection of skin tissues from oxidation processes. Figure 2 shows the antioxidant activity of the compound tested, verified by two methods DPPH and ABTS. The data obtained clearly confirm that all new triterpenes exhibit better antioxidant activity comparing to lupeol. The most effective lupeol ester, lupeol isonicotinate, neutralized 27.66 and 29.96% of DPPH and ABTS radicals, respectively, while lupeol just 1.40 and 0.64%. The standard antioxidant—gallic acid exhibited 89.90% and 97.24% of inhibition, respectively for both methods.

Studies made by Choon Young et al., confirm the influence of electron donating groups present in a molecule on its antioxidant activity [22]. The antioxidant assays that evaluate the direct effectiveness of compounds against free radical species can be divided into two main reaction categories, depending on the mechanism involved: Hydrogen Atom Transfer (HAT) and Single Electron Transfer (SET). The ABTS and DPPH assays show clearly that the compounds tested act according to the HAT mechanism, being able to reduce ROS by a single hydrogen atom, unlike the SET mechanism where free radicals are neutralized by a single electron [23,24]. The modification of the lupeol molecule with carboxylic acid or its anhydride influences the total antioxidant activity of the final ester.

Figure 3 shows the results of the evaluation of lupeol esters antioxidant activity according to FRAP method. In this case, the mechanism of the antioxidant activity is related to SET mechanism. The mechanism detects the ability of a potential antioxidant to transfer one electron to reduce any compound with free radical character [23].

The results showed in Figure 3 are compatible with the previous ones obtained using DPPH and ABTS methods. The most effective antioxidant was lupeol isonicotinate (0.59 FRAP units) and there was a significant increase in antioxidant activity of the lupeol derivatives, compared to the parental lupeol. The modification of lupeol structure influenced the ability of the obtained compounds to reduce free radical forms by two different mechanisms SET and HAT. However, when comparing to standard antioxidant like gallic acid, (3.10 FRAP units), the ability of neutralizing free radical forms for lupeol and its derivatives is relatively low. This allows the conclusion that the reduction of free radicals is not the main mechanism of antioxidant activity of the triterpenes. Indirect antioxidant activity was examined as an alternative mechanism for preventing skin damage by reactive oxygen species.

#### 3.1.2. Capability to Prevent Structural Damage of Proteins

The chemically induced oxidation performed with SLS, allowed the determination of the level of protection of tested compounds for the protein structure. The assay was conducted basing of the concentration of free thiol and carbonyl groups in albumin structure. The content of thiol and carbonyl groups reflects the level of protein degradation [19]. All experiments were compared to the control sample (without compounds tested) with the maximum level of protein structure decomposition. Figure 4 presents the results obtained.

The results presented in Figure 4 shows that there is a visible effect of lupeol esters on the protein protection from oxidation. The level of protein structure protection is comparable to glutathione. Comparing the results obtained for samples containing lupeol esters to the control samples which were no treated with the triterpenes or glutathione we observe a significant difference showing that the compounds inhibit the oxidation process of protein structure. The concentrations of thiol groups in the case of samples containing lupeol esters were about 97–98% lower than in samples without the actives. In case of carbonyl groups, the concentrations were about 86–98% lower. Stadtman in his studies shows that there is a correlation between aging processes and the accumulation of proteins damaged by oxidation [4]. The values obtained in our study show unequivocally that lupeol derivatives effectively prevent protein denaturation. Importantly, the lupeol ester’s activity was comparable to glutathione which was used in the study as the standard antioxidant (the concentrations of thiol and carbonyl groups: 0.0027 and 0.0012 mmol/dm^3^, respectively). The results obtained show that, in the case of lupeol esters, the major antioxidant activity mechanism is based on the protection of protein structure from free radical damage. Furthermore, the combination of two mechanisms, both the protein protection activity, with the ability for neutralizing free radicals, makes the compounds multifunctional antioxidants. In the human organism, with age, the level of glutathione (the main antioxidant) decreases causing the process of degradation of skin proteins [25]. Based on these results, it could be stated that the lupeol derivatives obtained can be successfully applied in anti-age cosmetics as glutathione supplements.

### 3.2. The Effect on the Proliferation and Migration of Human Skin Cells In Vitro

Apart from antioxidant activity, the influence of active compounds on epidermal tissues, especially on the viability, proliferation and migration of skin cells, is important when considering their use in skin damage therapy as well as in skin care. Cell viability assay is crucial in the safety evaluation of the compounds used for the skin, especially when skin barrier is damaged and exposed to external factors like harmful environmental conditions. Our previous research presented the influence of these compounds on the viability of human fibroblasts and keratinocytes [13].

No cytotoxic effects were observed for both keratinocytes and fibroblasts after incubation with tested compounds for 24 and 48 h.

These results indicate clearly that the lupeol esters exhibit no negative effect on the viability of skin cells in vitro at the same concentrations as used in the cell proliferation and migration assays presented in this study.

#### 3.2.1. Cell Proliferation

**Figure 5:**
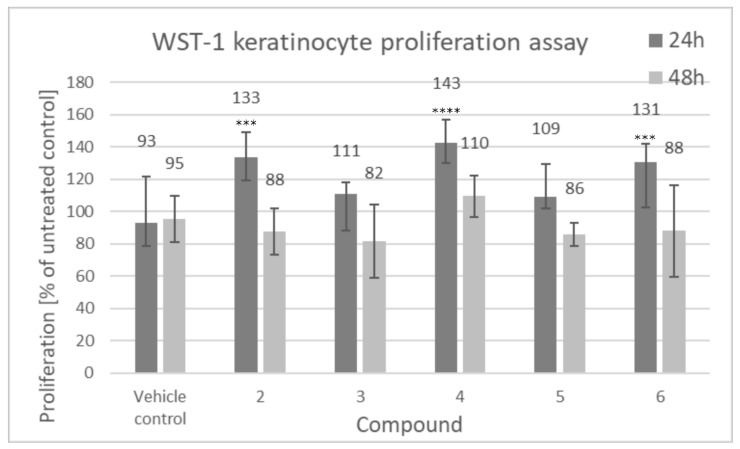
Proliferation of human keratinocytes (WST-1 assay), compounds tested at 100 µM (2: lupeol acetate, 3: lupeol propionate, 4: lupeol isonicotinate, 5: lupeol succinate, 6: lupeol acetylsalicylate). Cells were seeded at the density of 5 × 10^3^ cells per well of a 48-well plate, 24 h prior to addition of tested compounds. The incubation was carried out for 24 h and 48 h. Data presented as mean values ± SD. Statistical analysis by multiple comparison one-way ANOVA; *** *p* ≤ 0.001; **** *p* ≤ 0.0001 compared with the vehicle control.

Figure 5 shows proliferative activity of human keratinocytes, calculated relative to the individual donor untreated control and then averaged from 3 biological replicates.

As shown in Figure 5, an increase in keratinocyte mitochondrial activity, proportional to the total number of cells, after 24 h of incubation with the tested compounds was observed. It was detected that lupeol isonicotinate (4), lupeol acetate (2) and lupeol acetylsalicylate (6) were the most active compounds, which induced cell proliferative activity (133%, 143% and 131%, respectively) after 24 h of incubation compared to the untreated control sample. These three compounds have been subjected to more detailed studies presented in this work. This effect seems to be, however, transient as no significant effects on the proliferation of cells were observed after 48 h incubation with the compounds.

**Figure 6:**
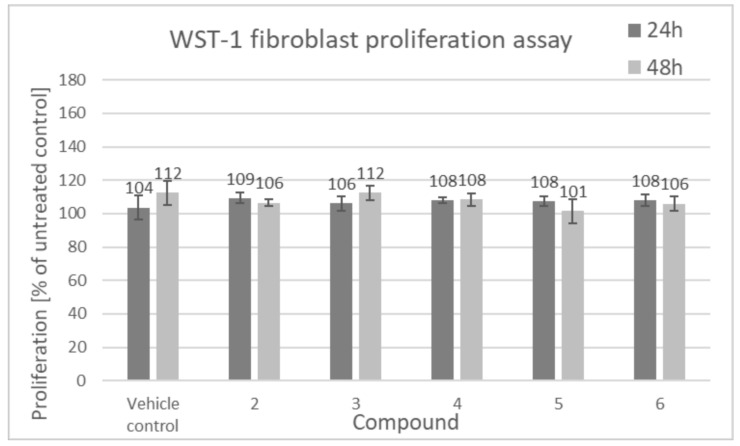
Proliferation of human dermal fibroblasts (WST-1 assay), compounds tested at 100 µM (2: lupeol acetate, 3: lupeol propionate, 4: lupeol isonicotinate, 5: lupeol succinate, 6: lupeol acetylsalicylate). Cells were seeded at the density of 5 × 10^3^ cells per well of a 48-well plate, 24 h prior to addition of tested compounds. The incubation was carried out for 24 h and 48 h. Data presented as mean values ± SD.

Figure 6 shows human dermal fibroblast proliferation, calculated relative to the individual donor untreated control and then averaged from 3 biological replicates.

As shown in Figure 6, there was no significant influence of the compounds on fibroblast proliferation. Akin to our results, Ebeling et al., demonstrated that there were no proliferative effects on fibroblasts induced by triterpene extract, betulin, lupeol and betulinic acid [1].

#### 3.2.2. Time Lapse Analysis of Movement of Individual Cells

**Figure 7:**
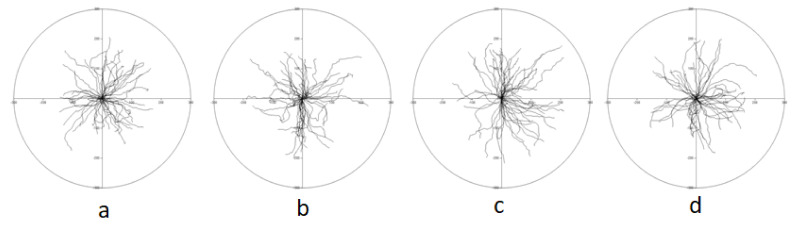
Diagrams representing keratinocyte motility. The starting point of individual cell trajectory is in the center of the diagram; vehicle control (**a**), lupeol acetate (**b**), lupeol isonicotinate (**c**) and lupeol acetylsalicylate (**d**). Cells were seeded 4 days before adding compounds at the density of 5 × 10^4^ cells per well of a 6-well plate. Cell traffic was recorded for 1.5 h, 90-sec step in time, starting 30 min after the addition of tested compounds (100 µM). Average values from each of 3 patients are presented (50 keratinocytes per patient).

As the directional cell migration is a crucial step during wound healing [26], the influence of lupeol esters on keratinocyte motility was determined, using time-lapse monitoring of the movement of individual cells, as well as in a wound healing assay in vitro (scratch assay). Figure 7 presents the analysis of the motility of individual keratinocytes after incubation with lupeol esters.

Movement parameters of keratinocyte motility after the incubation with lupeol acetate, isonicotinate and acetylsalicylate are summarized in Table 1.

Among all the tested compounds, lupeol isonicotinate had an effect accelerating keratinocyte motility. When compared to the vehicle control, average speed (*p* = 0.009), distance (the total length of cell trajectory; *p* = 0.009), the total length of cell displacement (*p* = 0.007) and average speed of cell displacement (*P* = 0.007) were significantly increased.

#### 3.2.3. Scratch Assay

**Figure 8:**
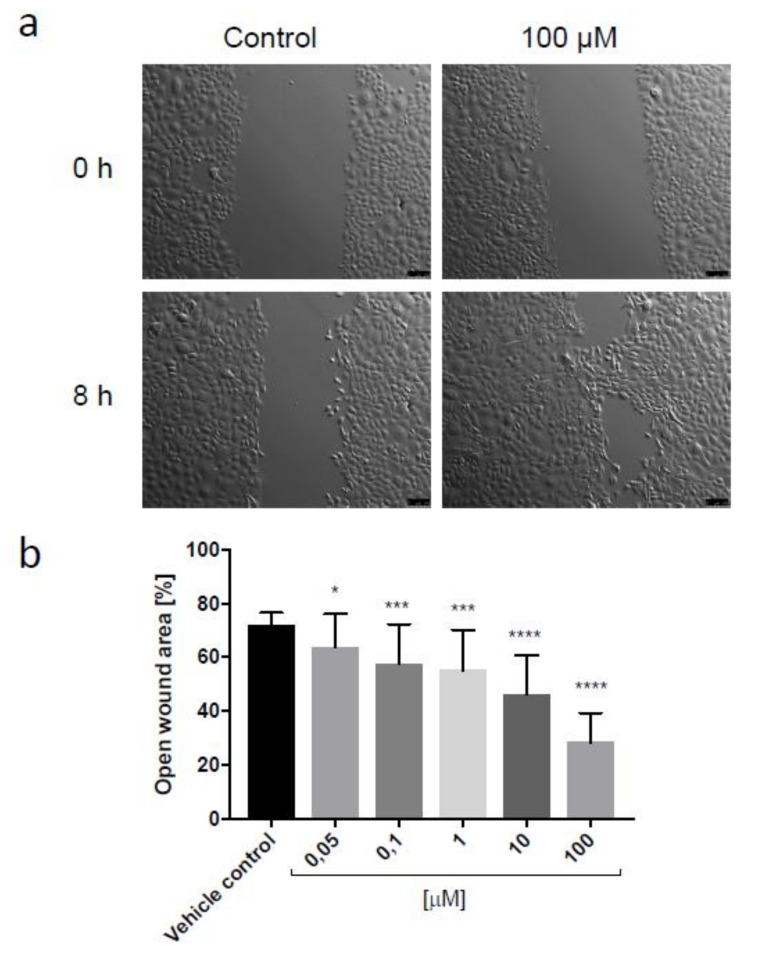
Representative wound closure images (scratch assay) of keratinocytes incubated in the presence of 100 µM lupeol isonicotinate or vehicle control, immediately after the scratches had been made and after 8 h. Scale bar = 100 µm (**a**). Relative open wound area (**b**); data presented as mean values ± SD from two independent experiments, each bar corresponds to 15–18 wound fields of view. Statistical analysis by multiple comparison one-way ANOVA: * *p* ≤ 0.05; *** *p* ≤ 0.01; **** *p* ≤ 0.0001 compared with the vehicle control.

Ebeling et al. demonstrated that birch bark extract containing lupeol influences the second stage of wound healing, the new tissue formation phase, by increasing cell migration of primary human keratinocytes shown in a scratch assay experiment. However, in the case of the birch bark extract, only keratinocytes migration seems to be affected, since we detected no visible increase in the number of proliferative cells [1]. Taking into account the positive effect of lupeol isonicotinate on the motility of keratinocytes in a short-term experiment and the increase in proliferative activity of keratinocytes incubated with this compound for 24 h, as well as its ability to reduce free radicals in antioxidant tests, lupeol isonicotinate was indicated as a compound showing particularly high activity. Therefore, this molecule was chosen for further testing in a wound healing assay in vitro. Figure 8 presents the representative wound closure images of keratinocytes migrating in the presence of 100 µM lupeol isonicotinate and vehicle control, as well as the analysis of relative open wound area for various concentrations of lupeol isonicotinate applied in the experiment.

Lupeol isonicotinate significantly accelerated wound closure in the scratch assay, in a dose-dependent manner. Concentration as low as 0.1 µM promoted migration of keratinocytes in vitro. Stimulation of the motility of epidermal cells can contribute to the improvement of the regeneration of the epidermal barrier and its function.

#### 3.2.4. Cell Morphology and Cytoskeleton

According to Ebeling et al., an influence on cell motility, measured by rearrangement of the actin cytoskeleton, could be observed for lupeol [1]. Interestingly, the reorganization of the actin cytoskeleton in terms of controlled polymerization and depolymerization is involved in cell migration. It provides a driving force for keratinocytes, at the wound edge, which results in wound closure. Filopodia, lamellipodia and stress fibers are various kinds of actin structures, which contribute to cell motility [27,28]. Basing on this, the keratinocytes and fibroblasts morphology and cytoskeleton was evaluated after the exposure to lupeol derivatives. We have focused on three the most potential compounds: lupeol acetate, lupeol isonicotinate and lupeol acetylsalicylate. Detailed experiments allowed to evaluate the mechanism of the triterpenes activity, especially their influence on cells migration.

**Figure 9:**
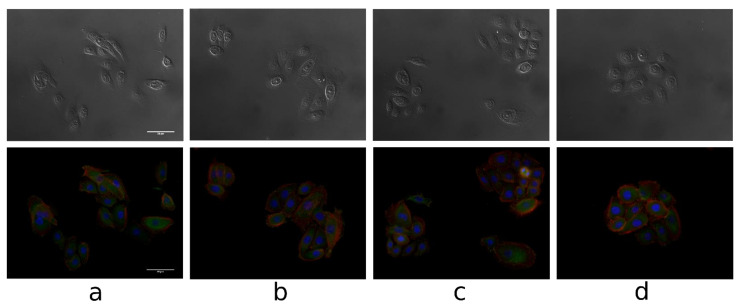
The fluorescence images of keratinocytes after 24 h of incubation with tested compounds (100 uM): control medium (**a**) lupeol acetate (**b**), lupeol isonicotinate (**c**) and lupeol acetylsalicylate (**d**). Scale bar = 500 µm, red color—actin fibers, green color—vinculin, blue color—cell nuclei (Hoechst Stain solution).

Figure 9 presents the microscopic images of keratinocytes after 24 of incubation with tested compounds.

As showed on the above pictures, it can be stated that both, lupeol acetate, isonicotinate and acetylsalicylate affect the formation of the actin fibers. The actin-rich region became more visible after incubation with all of the mentioned triterpenes. The cell nuclei as well as vinculin remained unaffected. The creation of actin fibers may result in the formation of lamellipodia and filopodia which supports cells migration. The literature studies claim that even low concentrations of triterpenes extracts induced the formation of filopodia and lamellipodia as well as stress fibers to a greater extent compared to the higher concentrations [1].

**Figure 10:**
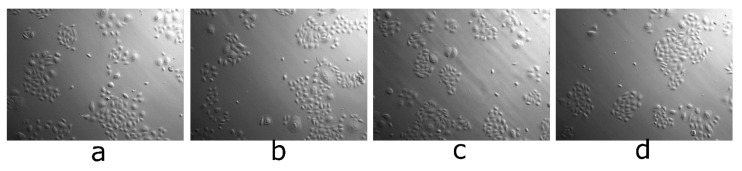
The impact of the tested compounds (100 uM) on cellular morphology for keratinocytes: control medium (**a**) lupeol acetate (**b**), lupeol isonicotinate (**c**) and lupeol acetylsalicylate (**d**), 100× magnification, 36l K cells (2p), Cells were seeded 24 h before adding compounds, at the density of 5 × 10^4^ cells per well of a 6-well plate, picture made after 2 h of incubation with compounds.

Figure 10 presents the microscopic images of keratinocytes morphology after 2 h of incubation with tested compounds.

The incubation of keratinocytes with lupeol acetate, isonicotinate and acetylsalicylate did not significantly affect cell morphology. There were no changes observed compared to the control sample.

**Figure 11:**
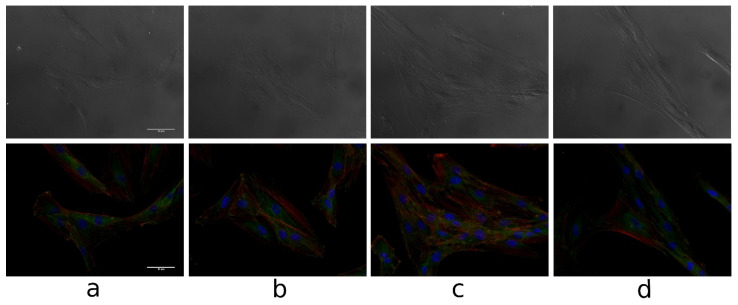
The microscopic images and the fluorescence microscopic images of fibroblasts after 24 h incubation with tested compounds (100 uM): control medium (**a**) lupeol acetate (**b**), lupeol isonicotinate (**c**) and lupeol acetylsalicylate (**d**). Scale bar = 500 µm, 36l K cells (B) 3p, red color—actin fibers, green color—vinculin, blue color—cell nuclei (Hoechst Stain solution).

Figure 11 presents the microscopic images and the fluorescence microscopic images of fibroblasts after 24 of incubation with tested compounds.

Visible actin fibers can be observed and there is a significant increase in the fibers formation in compare to the control culture, especially in the cells incubated with lupeol acetate and lupeol isonicotinate. The results obtained are consistent with those obtained for keratinocytes. Vinculin and cell nuclei structures are unchanged. While the migration of fibroblasts and keratinocytes is of great importance in the wound healing process, keratinocytes motility is crucial.

## 4. Conclusions

The aim of this study was to evaluate the potential of new triterpene compounds, lupeol esters, as active ingredients dedicated for skin barrier recovery. Lupeol esters are confirmed to possess bifunctional antioxidant activity. They can play a dual protective role: scavenging free radicals by two different mechanisms and protecting protein structure against damage in the oxidative environment. Therefore, the molecules act multi-directionally to resolve the consequences of skin barrier damage and ensure long-term protection against hazardous conditions and accelerated skin aging process. According to the results obtained, lupeol isonicotinate was the most effective antioxidant in reducing free radicals. Importantly, all of the triterpenes were effective in protecting proteins from oxidation as their activity is comparable to glutathione. Given that lupeol had already shown promising wound healing properties in patients [10,11] the observed increase in antioxidant activity may be beneficial in wound healing.

In our study, there was no significant effect on the proliferation of both keratinocytes and fibroblasts, however, a transient increase in proliferative activity of keratinocytes was observed for cells treated for 24 h with lupeol isonicotinate, lupeol acetate and lupeol acetylsalicylate. Lupeol isonicotinate was the most potent compound in these experiments. What is more, lupeol isonicotinate promoted the motility of keratinocytes in the time-lapse monitoring of movement of individual cells, as well as accelerated wound healing in the scratch assay. Obtained data indicate the possibility of using lupeol isonicotinate as a new therapeutic skin care ingredient, supporting the natural mechanisms of skin reconstruction. Our results show that novel lupeol esters can be involved in the wound healing process via multiple mechanisms of activity.

## Figures and Tables

**Table 1:** Movement parameters of cells; time-lapse monitoring of individual cell motility (2: lupeol acetate, 4: lupeol isonicotinate, 6: lupeol acetylsalicylate). Data presented as mean values ± SD. Statistical analysis by unpaired Student’s t-test compared with the vehicle control. CDE = coefficient of dislocation efficiency (the ratio of cell displacement to the length of cell trajectory).

**Condition:**	**Vehicle Control**	**2**	**4**	**6**
Speed [µm/min]:	1867 ± 0.060	1893 ± 0.074	2104 ± 0.067	1947 ± 0.061
Distance [µm]:	168,004 ± 5364	170,403 ± 6681	189,395 ± 6006	175,209 ± 5515
Displacement [µm]:	135,875 ± 6157	132,519 ± 7250	159,458 ± 6136	140,483 ± 6639
Speed displacement [µm/min]	1510 ± 0.068	1472 ± 0.081	1772 ± 0.068	1561 ± 0.074
CDE:	0.797 ± 0.025	0.763 ± 0.026	0.836 ± 0.013	0.792 ± 0.026

## Data Availability

Not applicable.
